# Distinct inflammatory phenotypes of microglia and monocyte‐derived macrophages in Alzheimer's disease models: effects of aging and amyloid pathology

**DOI:** 10.1111/acel.12522

**Published:** 2016-10-08

**Authors:** Elodie Martin, Céline Boucher, Bertrand Fontaine, Cécile Delarasse

**Affiliations:** ^1^ Inserm U 1127CNRS UMR 7225Sorbonne UniversitésUPMC Univ Paris 06 UMR S 1127Institut du Cerveau et de la Moelle épinière (ICM)F‐75013ParisFrance; ^2^ AP‐HPHôpital de la Pitié SalpêtrièreF‐75013ParisFrance

**Keywords:** aging, Alzheimer's disease, chemokines, cytokines, macrophages, microglia

## Abstract

Alzheimer's disease (AD) is a neurodegenerative disease characterized by formation of amyloid‐β (Aβ) plaques, activated microglia, and neuronal cell death leading to progressive dementia. Recent data indicate that microglia and monocyte‐derived macrophages (MDM) are key players in the initiation and progression of AD, yet their respective roles remain to be clarified. As AD occurs mostly in the elderly and aging impairs myeloid functions, we addressed the inflammatory profile of microglia and MDM during aging in TgAPP/PS1 and TgAPP/PS1dE9, two transgenic AD mouse models, compared to WT littermates. We only found MDM infiltration in very aged mice. We determined that MDM highly expressed activation markers at basal state. In contrast, microglia exhibited an activated phenotype only with normal aging and Aβ pathology. Our study showed that CD14 and CD36, two receptors involved in phagocytosis, were upregulated during Aβ pathogenesis. Moreover, we observed, at the protein levels in AD models, higher production of pro‐inflammatory mediators: IL‐1β, p40, iNOS, CCL‐3, CCL‐4, and CXCL‐1. Taken together, our data indicate that microglia and MDM display distinct phenotypes in AD models and highlight the specific effects of normal aging *vs* Aβ peptides on inflammatory processes that occur during the disease progression. These precise phenotypes of different subpopulations of myeloid cells in normal and pathologic conditions may allow the design of pertinent therapeutic strategy for AD.

## Introduction

Alzheimer's disease (AD) is a neurodegenerative disease that mainly affects the elderly. The histopathologic hallmarks of AD are senile plaques composed of extracellular aggregates of amyloid‐β (Aβ) peptides and neurofibrillary tangles. These pathologic changes appear with inflammation well established by histology. Aβ plaques are surrounded by many activated myeloid cells and astrocytes (Heneka *et al*., 2010). Genetic studies have identified genes associated with innate immune functions suggesting that myeloid cells could contribute significantly to the pathogenesis of AD (Lambert *et al*., 2010).

Myeloid cells play an important role in the homeostasis of the CNS, and their properties are altered in aging (Harry, 2013). The immune response that occurs during normal aging and AD displays analogies, but their specificities remain to be identified (Heneka *et al*., 2010; Mosher & Wyss‐Coray, 2014). Thus, it is critical to discriminate whether normal aging processes alter myeloid cells functions and could contribute to Aβ deleterious responses or whether the excess of Aβ accumulation by itself induces changes of phenotype of myeloid cells to a pro‐inflammatory profile that promotes AD progression (Conde & Streit, 2006). Identification of specific effect of each immune pathway would allow to determine which target should be modulated in AD.

The two main myeloid cells of the CNS are resident microglia that derive from yolk sac progenitors during embryogenesis and monocyte‐derived macrophages (MDM) that differentiate from bone marrow progenitors and can infiltrate CNS during brain injury. Myeloid cells have dual roles in AD processes depending on their activation state. These cells can release pro‐inflammatory mediators or be beneficial through phagocytosis of Aβ peptides. Experimental models using bone marrow chimeric mice or microglial ablation systems have shown that microglia appear to have a deficient role in AD with motility and phagocytic activities impaired (Grathwohl *et al*., 2009; Krabbe *et al*., 2013), while MDM seem to contribute more efficiently to the elimination of Aβ plaques (Simard *et al*., 2006). In contrast, in experimental autoimmune encephalomyelitis (EAE), an animal model of multiple sclerosis, infiltration of activated MDM is involved in the initiation of the demyelinating processes, while microglia contribute to remyelination (Ajami *et al*., 2011; Yamasaki *et al*., 2014). In AD models, the characteristics of MDM and microglia still need to be clearly defined in more physiologic conditions without experimental recruitment of MDM to find pathways to regulate them.

To determine the precise phenotypes and activation states of microglia and MDM in response to Aβ peptides or due to normal aging, we choose the TgAPP/PS1 model with early‐onset Aβ plaque formation to highlight an effect of Aβ accumulation before aging processes (Radde *et al*., 2006), and we confirmed our data using a less aggressive model TgAPP/PS1dE9 (Jankowsky *et al*., 2004). We also compared the phenotype of MDM and microglia in AD model and in the well‐characterized model EAE to identify the phenotype associated with beneficial functions. We found that a MDM population was recruited to Aβ plaques and that MDM infiltration occurs only during aging and at late stage of Aβ pathology. Taken together, our data indicate that microglia and MDM exhibited distinct phenotypes and were differentially activated by normal aging and Aβ peptides. Our study highlights that the immune response to Aβ plaques was mainly characterized by persistent expression of several pro‐inflammatory mediators.

## Results

### The proportion of the MDM population is increased during aging in WT mice and to a greater extent in old AD models

To study the role of microglia and MDM in TgAPP/PS1 and TgAPP/PS1dE9, two AD models, we first analyzed Aβ plaques at different disease stages. TgAPP/PS1 mice exhibited an early appearance of Aβ plaques since 4 months of age (Fig. S1A), while TgAPP/PS1dE9 mice exhibited very few plaques at 6 months (Fig. S1B).

**Figure 1:**
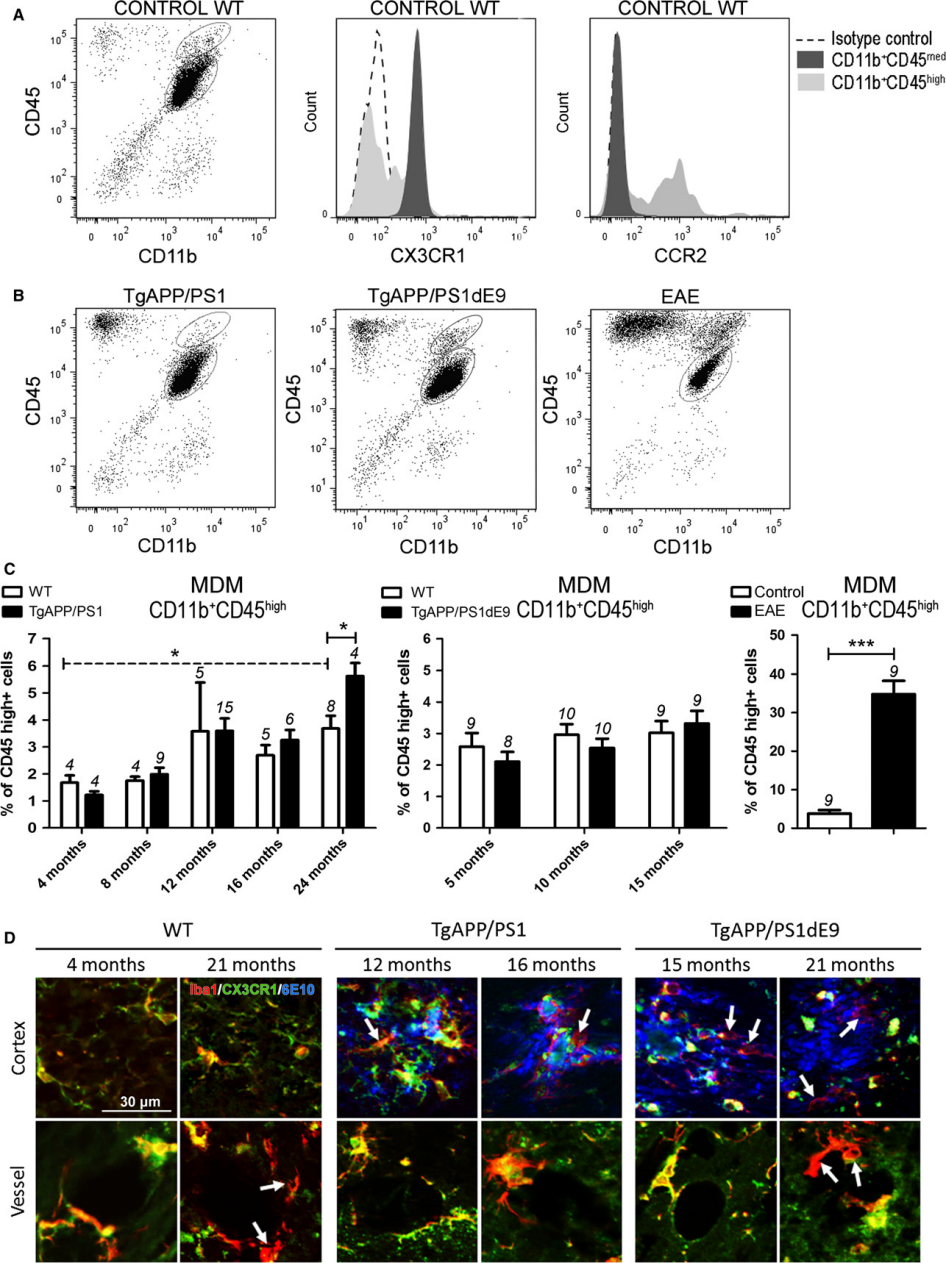
Analyses of microglia and MDM populations in two AD models and EAE mice. (A) FACS analysis of microglia and MDM in the brain of WT controls (16 months). Brain cells were stained for CD45 and CD11b. Gates shown in the dot plots illustrate CD11b^+^ CD45^med^ microglia population and CD11b^+^ CD45^high^ MDM population (left panel). FACS histograms (middle and right panels) show CX3CR1 and CCR2 expression in microglia (CD11b^+^ CD45^med^) and MDM populations (CD11b^+^ CD45^high^). Isotype antibodies were used as negative controls (dotted histogram). (B) FACS analysis of microglia and MDM in the brain of TgAPP/PS1 mice (16 months), TgAPP/PS1dE9 mice (15 months), and EAE mice. Brain cells were stained for CD45 and CD11b. Gates shown in the dot plots illustrate CD11b^+^ CD45^med^ microglia population and CD11b^+^ CD45^high^ MDM population. (C) Histograms indicate the percentage of CD11b^+^ CD45^high^ from total CD11b+ cells at different ages in WT littermate controls, TgAPP/PS1 mice, TgAPP/PS1dE9 mice, and EAE mice. The number of mice is indicated above each histogram bar. (D) Brain sections were stained for myeloid cells with anti‐Iba1 antibody (red), microglia with anti‐CX3CR1 antibody (green), and Aβ plaques with anti‐Aβ antibody (6E10) (blue). MDM cells are indicated by an arrow. Data are expressed as means ± SEM. *P < 0.05. ***P < 0.001 statistical significance.

The number of Aβ‐associated myeloid cells was shown to be increased in both models with the disease progression (Radde *et al*., 2006; Hickman *et al*., 2008) and restricted to the halo of cored plaques (6E10^+^/thioflavin^−^) (Fig. S1C, D). Microglia and MDM can be distinguished by their expression profiles by flow cytometry (FACS): CD11b^+^CD45^med^ for microglia and CD11b^+^CD45^high^ for MDM (Sedgwick *et al*., 1991). It has also been reported that microglia express CX3CR1 but not CCR2 marker in contrast to MDM (Mizutani *et al*., 2012). As expected, we observed that all microglial cells (CD11b^+^CD45^med^ gate) were CX3CR1^+^CCR2^−^ while some MDM (CD11b^+^CD45^high^ gate) expressed CCR2 (Fig. 1A). It was interesting to note that a MDM population was already present in WT mice, in nonpathologic conditions (Fig. 1A). We next analyzed microglia and MDM populations in the two AD models and in EAE, the well‐established animal model of MS in which MDM infiltration was clearly described (Ajami *et al*., 2011). We decided to study EAE mice at disease score of 3 when a maximal infiltration of MDM occurs (Fig. 1B). To assess the contribution of MDM in AD compared to WT mice and during normal aging, we analyzed the proportion of MDM in the myeloid population (CD11b^+^) (Fig. 1B). We found that the proportion of MDM was increased in the brain of TgAPP/PS1 mice compared to WT mice only at late stage of the disease (Fig. 1C), whereas it was strongly increased (9.1‐fold) in the brains of the inflammatory disease EAE compared to control mice (Fig. 1C). Interestingly, we observed an effect of normal aging on the proportion of MDM in the brain (Fig. 1C).

By histology, we identified several Iba1 + CX3CR1^neg^ cells in the corpus callosum and around vessels in EAE mice corresponding to MDM infiltration (Fig. S2). In both AD models, we observed few Iba1 + CX3CR1^neg^ cells around Aβ plaques at advanced disease stages (Figs. 1D and S2). We only detected perivascular MDM in aged WT mice and at late stage of the disease in 21‐month‐old TgAPP/PS1dE9 but not at less advanced stages: 16‐month‐old TgAPP/PS1 and 15‐month‐old TgAPP/PS1dE9 (Figs. 1D and S2).

In conclusion, our data indicate that the proportion of MDM is increased in aged mice and suggest that MDM infiltration is a late process during the Aβ pathology as observed in sharp contrast with EAE.

### Microglia exhibited an activated phenotype only in Aβ pathology and with aging, while MDM were activated at basal state

**Figure 2:**
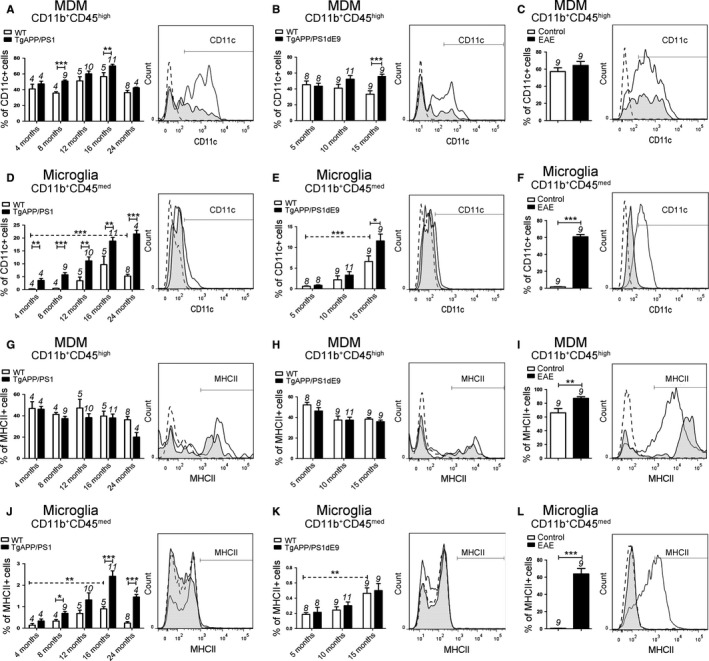
Analyses of microglia and MDM activation states in two AD models and EAE mice. CD11c (A–F) and MHCII (G–L) expression on CD11b^+^ CD45^high^ MDM populations (A–C and G–I) and CD11b^+^ CD45^med^ microglia (D–F and J–L) from WT littermate controls, TgAPP/PS1 mice (A, D, G, J), TgAPP/PS1dE9 mice (B, E, H, K), and EAE mice. Gates shown in the FACS histograms (right panel) illustrate CD11c (A–F) and MHCII (G–L) expression in CD11b^+^ CD45^high^ cells and CD11b^+^ CD45^med^ cells from WT mice (black histogram) and 16‐month‐old TgAPP/PS1 mice (A, D, G, J), 15‐month‐old TgAPP/PS1dE9 mice (B, E, H, K), and EAE mice (C, F, I, L) (black tinted histogram). Isotype antibodies were used as negative controls (dotted histogram). The number of mice is indicated above each histogram bar. Data are expressed as means ± SEM. *P < 0.05, **P < 0.01, ***P < 0.001 statistical significance.

To determine the activated phenotype of both myeloid populations during normal aging and in AD models, we analyzed in microglia *vs* MDM the expression of MHCII and CD11c, two surface proteins involved in antigen presentation and known to be upregulated in activated myeloid cells (Fig. 2). First, we observed that MDM highly expressed the activation marker CD11c and MHCII at basal state in WT mice (Fig. 2A, B, G, H) in contrast to microglia (Figs. 2D, E, J, and K and 6).

We next analyzed the expression of CD11c and MHCII in myeloid cells in AD models compared to WT mice. The number of MDM expressing CD11c was increased in both AD models (Fig. 2A, B), whereas it was unchanged in EAE *vs* control mice (Fig. 2C). However, we observed that the percentage of MDM expressing MHCII was unchanged in AD models compared to WT mice (Fig. 2G, H), whereas it was increased in EAE compared to control mice (Figs. 2I and 6).

Importantly, a significant increase in microglia expressing CD11c was observed in AD models compared to WT mice (Fig. 2D, E) and in EAE *vs* control mice (Fig. 2F). In contrast to MDM, we found that expression of MHCII in microglia was increased in 8‐ and 16‐month‐old TgAPP/PS1 compared to age‐matched WT littermate (Fig. 2J, K), and greatly upregulated in EAE compared to control mice (Figs. 2L and 6).

To assess the effect of normal aging on activation state of myeloid cells, we also analyzed the expression of MHCII and CD11c in young and old WT mice. We observed that expression of CD11c and MHCII in MDM was not modified during normal aging (Figs. 2A, B, G, and H and 6). In contrast, the expression of CD11c and MHCII was significantly increased in microglia with the age of the animals in WT mice (Figs. 2D and J and 6).

Our data show that MDM are highly immune activated at basal state in contrast to microglia that are only activated during normal aging and Aβ pathologic processes (Fig. 6).

### Coreceptors of phagocytosis were strongly expressed in WT MDM, while microglia exhibited little baseline expression but increased expression in AD models

Myeloid cells are involved in the clearance of Aβ plaques through the phagocytosis of Aβ peptides. To determine whether aging and Aβ deposition modify the expression of proteins involved in phagocytic activity in myeloid cells, we analyzed the expression of coreceptors of pattern recognition receptors. These receptors are able to mount an immediate immune response to pathogens or danger signals such as Aβ structure. The Toll‐like receptors 2/4 (TLRs), coreceptor CD14, and the scavenger receptor CD36 contribute to recognition and binding of fibrillary Aβ by myeloid cells in the brain and are involved in the phagocytosis of Aβ peptides (Liu *et al*., 2005; Reed‐Geaghan *et al*., 2009; Stewart *et al*., 2010; Yamanaka *et al*., 2012).

**Figure 3:**
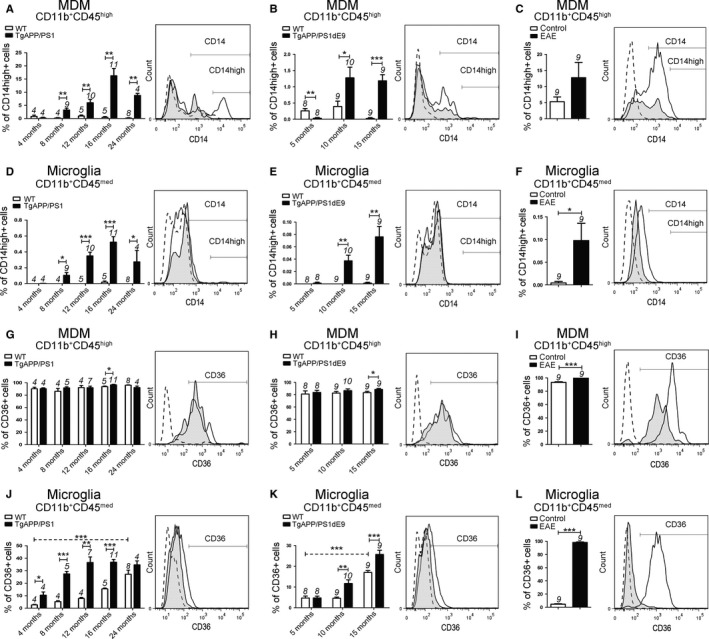
Analyses of CD14 and CD36 expression in microglia and MDM in two AD models and EAE mice. CD14 (A–F) and CD36 (G–L) expression on CD11b^+^ CD45^high^ MDM populations (A–C and G–I) and CD11b^+^ CD45^med^ microglia (D–F and J–L) from WT littermate controls, TgAPP/PS1 mice (A, D, G, J), TgAPP/PS1dE9 mice (B, E, H, K), and EAE mice (C, F, I, L). Gates shown in the FACS histograms (right panel) illustrate CD14 (A–F) and CD36 (G–L) expression in CD11b^+^ CD45^high^ cells and CD11b^+^ CD45^med^ cells from WT mice (black histogram) and 16‐month‐old TgAPP/PS1 mice (A, D, G, J), 15‐month‐old TgAPP/PS1dE9 mice (B, E, H, K), and EAE mice (C, F, I, L) (black tinted histogram). Isotype antibodies were used as negative controls (dotted histogram). The number of mice is indicated above each histogram bar. Data are expressed as means ± SEM. *P < 0.05, **P < 0.01, ***P < 0.001 statistical significance.

First, we observed that most MDM expressed CD36 and highly expressed membrane CD14 (∼33%) and (FACS histograms; Fig. 3A, B, G, H) in contrast to microglia (∼2.7% CD36^+^ and ∼0.8% CD14^+^) (FACS histograms; Fig. 3D, E, J, K) in WT mice at basal state (Fig. 6).

We found that CD14 was upregulated in MDM AD models compared to WT mice (Fig. 3A, B) while no difference in CD14 expression was found in MDM in EAE mice (Fig. 3C).

Furthermore, we found elevated expression of CD14 in microglia in AD models (Fig. 3D, E) and in EAE compared to control mice (Fig. 3F). Importantly, the percentage of microglia expressing CD36 was strongly increased in both AD models *vs* WT mice (Fig. 3J, K) and most of microglia expressed CD36 in EAE compared to control mice (Figs. 3L and 6).

We also assessed the effect of normal aging on CD14 and CD36 expression in myeloid cells in young and old WT mice. We found that normal aging does not modify the expression of CD14 in WT animals neither in MDM (Fig. 3A, B) nor in microglia (Fig. 3D, E). The expression of CD36 was also not modified with the age in MDM (Fig. 3G, H). In contrast, the expression of CD36 was significantly increased in microglia with the age of the animals in WT mice (Figs. 3J and K and 6).

Taken together, our data show that MDM strongly express coreceptors involved in phagocytosis while microglia mainly express these proteins in pathologic conditions (Fig. 6). In particular, the vast majority of MDM express CD36, whereas microglia exhibit an important increase in CD14 and CD36 expression during the disease that correlates with the progression of Aβ deposition observed in both AD models (Fig. 1).

### Expression of pro‐inflammatory cytokines is specifically increased in response to Aβ lesions

Cytokines are involved in the regulation of inflammatory and immune responses in the CNS during AD and modulate Aβ‐induced neurodegeneration. Most studies on AD analyzed the amount of cytokines in the CNS at the transcriptional level. However, several cytokines are initially expressed as leaderless biologically inactive pro‐forms that are activated following cleavage such as IL‐1β, IL‐1α, and TNF‐α (Dinarello & Margolis, 1995). Therefore, to study the role of inflammatory molecules during aging and in AD models, we analyzed the expression of these molecules at the protein level.

**Figure 4:**
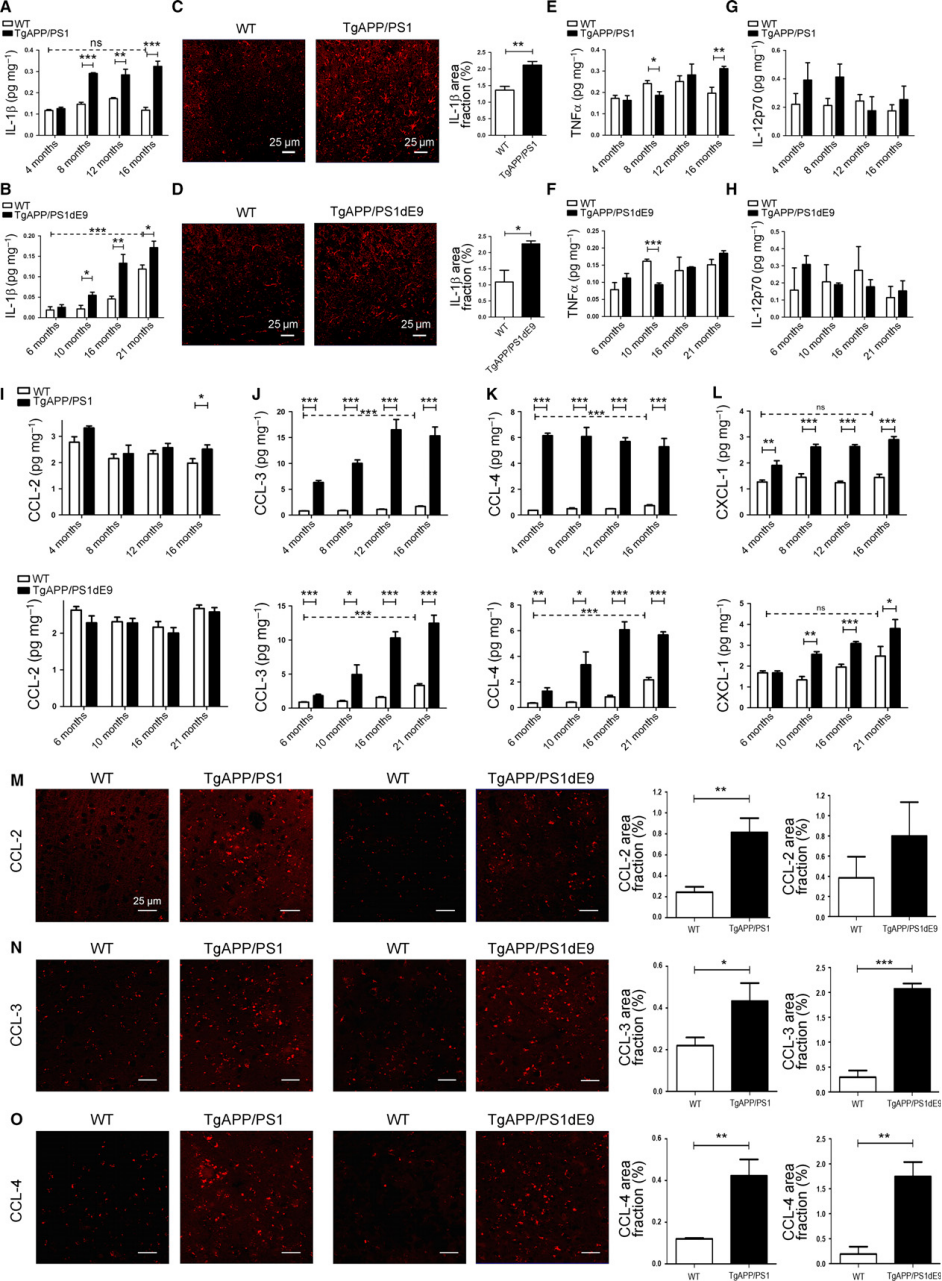
Analyses of cytokines and chemokines expression in two AD models. (A–B) IL‐1β protein expression levels were determined by multiplex ELISA in the brain of TgAPP/PS1 mice (A) and of TgAPP/PS1dE9 mice (B) and age‐matched WT littermate controls (*n* = 4‐6). (C–D) IL‐1β staining of brain slice of 16‐month‐old TgAPP/PS1 mice (C), 21‐month‐old TgAPP/PS1dE9 mice (D), and WT littermate controls. Quantification of surface area of IL‐1β staining was performed in 10 consecutive sections per animal and is given as % of area fraction (*n* = 5). TNF‐α (E–F) and IL‐12p70 (G–H) protein expression was determined by multiplex ELISA in the brain of TgAPP/PS1 mice (E–G) and of TgAPP/PS1dE9 mice (F, H) and age‐matched WT littermate controls (*n* = 4‐6). CCL‐2 (I), CCL‐3 (J), CCL‐4 (K), and CXCL‐1 (L) protein expression was determined by multiplex ELISA in the brain of TgAPP/PS1 mice (upper panel) and of TgAPP/PS1dE9 mice (lower panel) and age‐matched WT littermate controls (*n* = 4–6). CCL‐2 (M), CCL‐3 (N), and CCL‐4 (O) staining of brain slice of 16‐month‐old TgAPP/PS1 mice, 21‐month‐old TgAPP/PS1dE9 mice, and WT littermate controls (right panels); quantification of surface area of CCL‐2 (M), CCL‐3 (N), and CCL‐4 (O) (left panels). Staining was performed in 10 consecutive sections per animal and is given as % of area fraction (*n* = 5). Data are expressed as means ± SEM. *P < 0.05, **P < 0.01, ***P < 0.001 statistical significance.

The level of IL‐1β protein production was analyzed by ELISA in total brain extracts from AD models compared to WT mice at different stages corresponding to disease progression. We found an increase in IL‐1β production in brains from the age of 8 months in TgAPP/PS1 (Fig. 4A) and 10 months in TgAPP/PS1dE9 compared to WT (Fig. 4B). We also observed that IL‐1β level was increased in very old compared to young WT mice (6.4‐fold increase in 21‐month‐old *vs* 6‐month‐old) (Fig. 4B). To confirm our data, immunochemistry for IL‐1β was performed and analyzed in the cortex sections from older animals, 16‐month‐old TgAPP/PS1 and 21‐month‐old TgAPP/PS1dE9. The expression of IL‐1β was increased in the cortex of 16‐month‐old TgAPP/PS1 *vs* WT mice (Fig. 4C) and in 21‐month‐old TgAPP/PS1dE9 compared to WT mice (Figs. 4D and 6).

We also measured TNF‐α concentration in total brain extracts from AD models at different disease stages compared to WT mice. The TNF‐α brain concentration was reduced in 8‐month‐old TgAPP/PS1 mice (Fig. 4E) and 10‐month‐old TgAPP/PS1dE9 mice *vs* WT mice (Fig. 4F), whereas it was increased in the brain at latter stage of the disease in 16‐month‐old TgAPP/PS1 animals compared to WT mice (Fig. 4E). We next analyzed TNF‐α expression in MDM and microglia by FACS in AD models *vs* WT mice. We found a significant increase in TNF‐α level in MDM in 16‐ and 24‐month‐old TgAPP/PS1 and 10‐month‐old TgAPP/PS1dE9 mice (Fig. S3A, B), while no difference of TNF‐α expression was observed in microglia (Fig. S3D, E). TNF‐α expression level was not changed in microglia and MDM during EAE (Fig. S3C, F). In addition, we noticed a significant decrease in the percentage of TNF‐α‐expressing cells with aging but only in the MDM subpopulation (Fig. S3A, B, D, E).

We analyzed by FACS the expression of the p40 subunit of IL‐12 and IL‐23 in MDM and microglia in AD models and in EAE *vs* WT mice. A significant increase in p40 was observed in both cells in AD models compared to age‐matched WT mice (Fig. S3G, H, J, K). We also determined by ELISA the brain concentration of the p70 subunit of IL‐12 and found no difference in the brains of AD models *vs* WT mice at any age studied (Fig. 4G, H). These results suggested that IL‐23 level is increased rather than IL‐12 level in AD models (Fig. 6).

We performed multiplex ELISA to examine the overall production of several pro‐inflammatory (IL‐2, IL‐5, IL‐6, IFN‐γ) and anti‐inflammatory (IL‐4 and IL‐10) cytokines in brain extracts from AD models (Fig. S4). The use of two different AD models allowed us to highlight the effect of the Aβ pathology and not differences specific to a particular AD model as observed for IL‐4 and IL‐10 (Fig. S4D, E).

In summary, we observe modifications of cytokine production in response to the Aβ pathology but for the six cytokines tested, we did not observe a significant effect of normal aging (Fig. 6). The cytokines produced are mainly of the pro‐inflammatory repertoire suggesting that myeloid cells do not shift toward an anti‐inflammatory state with the disease progression.

### Chemokine expression is increased in AD models

Chemokines released in the CNS have been described as microglial chemoattractants and have been involved in the recruitment of myeloid progenitors from the periphery. In primary mouse microglia cultures, Aβ‐stimulation induces upregulation of several chemokine mRNAs including CCL‐2, CCL‐3, and CCL‐4 (El Khoury *et al*., 2003; Halle *et al*., 2008).

Here, we analyzed these chemokine concentrations in brain extracts from AD models. TgAPP/PS1 mice exhibited an increase in CCL‐2 production only at the age of 16 months compared to WT littermate, and no difference was observed in TgAPP/PS1dE9 mice *vs* WT animals at any age studied (Fig. 4I). We found a strong increase in CCL‐3 and CCL‐4 concentrations already at the early stages of Aβ deposition (Fig. 4J, K). The CCL‐3 and CCL‐4 concentrations were slightly increased with the age of the animals in WT mice (Fig. 4J, K). We also observed a significantly higher CXCL‐1 concentration in AD models *vs* WT mice that increased during the course of AD pathology (Figs. 4L and 6).

We confirmed these results by quantifying the immunostaining of CCL‐2, CCL‐3, and CCL‐4 in cortex sections at the latter stages of the pathology (Fig. 4M–O). We observed an increase in CCL‐2 expression in 16‐month‐old TgAPP/PS1 *vs* WT, whereas it was comparable in 21‐month‐old TgAPP/PS1dE9 *vs* WT (Fig. 4M), as observed by ELISA (Fig. 4I). The expression of CCL‐3 and CCL‐4 was strongly increased in the cortex of 16‐month‐old TgAPP/PS1 and of 21‐month‐old TgAPP/PS1dE9 compared to WT mice (Figs. 4N and O and 6).

Many different CNS cells have been identified as sources of chemokines. We observed that these chemokines were mostly expressed in GFAP‐positive astrocytes and were detected in few Iba‐1‐positive microglia and NeuN‐positive neurons (Fig. S5).

Our results show that chemokines are induced in both AD models and are mainly produced by astrocytes.

### Myeloid cells express iNOS around Aβ plaques

Using morphological markers, we observed an increase in the surface of Iba1 staining as well as the soma size of myeloid cells in TgAPP/PS1 compared to WT animals indicating an increase in myeloid cell activation (Fig. S6A, B).

**Figure 5:**
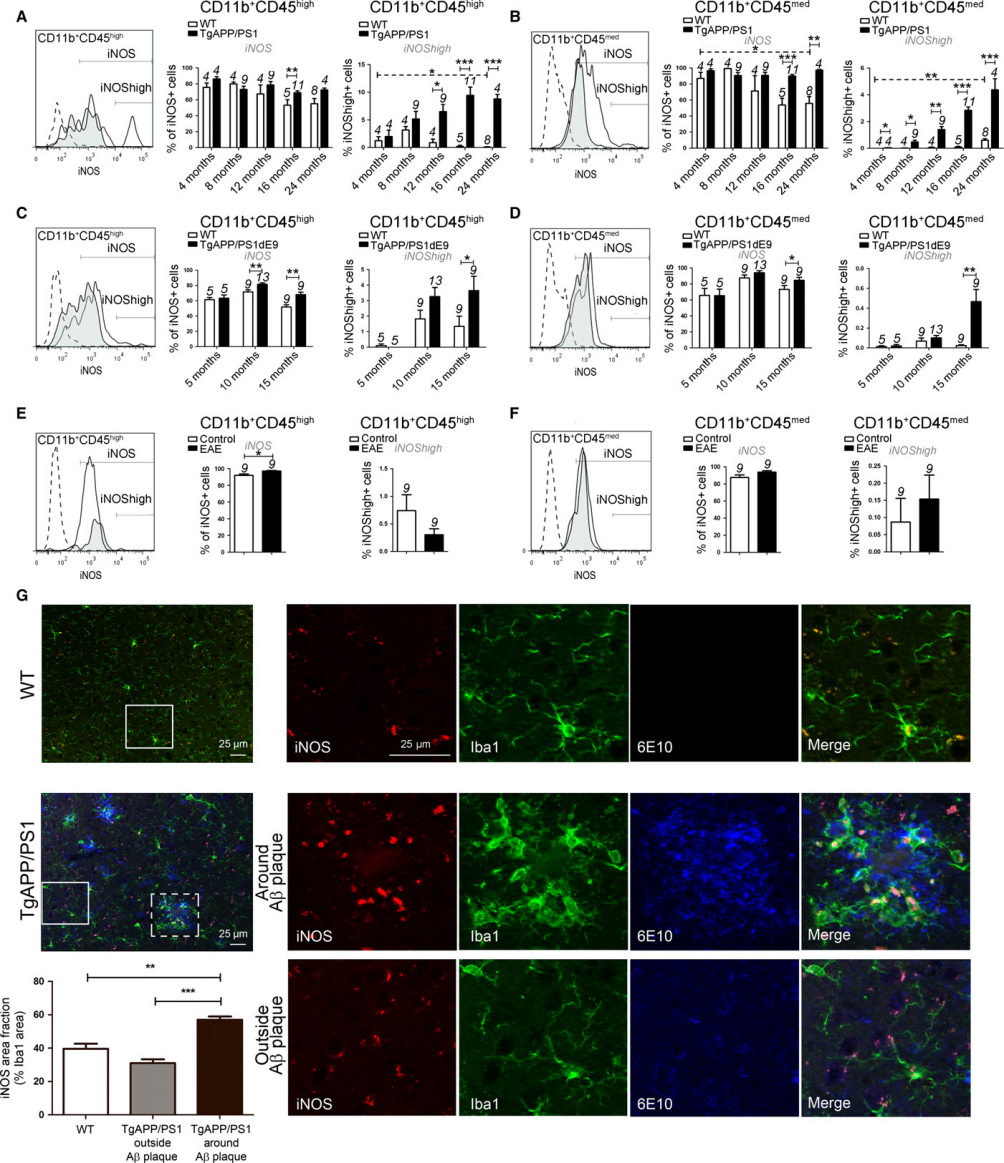
Analyses of iNOS expression levels in two AD models. iNOS expression on CD11b^+^ CD45^high^ MDM populations (A, C, E) and CD11b^+^ CD45^med^ microglia (B, D, F) from WT littermate controls, TgAPP/PS1 mice (A–B), TgAPP/PS1dE9 mice (C–D), and EAE mice (E–F). Gates shown in the FACS histograms (left panel) illustrate different iNOS expression levels in CD11b^+^ CD45^high^ cells (A, C, E) and CD11b^+^ CD45^med^ cells (B, D, F) from WT mice (black histogram), 16‐month‐old TgAPP/PS1 mice (A–B), 15‐month‐old TgAPP/PS1dE9 mice (C, D), and EAE mice (E–F) (black tinted histogram). Isotype antibodies were used as negative controls (dotted histogram). The number of mice is indicated above each histogram bar. (G) Brain sections of 16‐month‐old WT and TgAPP/PS1 mice were stained for iNOS (red), microglia with Iba1 antibody (green), and Aβ peptide with 6E10 antibody (blue). Quantification of surface area of iNOS staining on total Iba1 staining was performed in 10 consecutive sections per animal and is given as % of area fraction (*n* = 5 per group). Data are expressed as means ± SEM. *P < 0.05, **P < 0.01, ***P < 0.001 statistical significance.

Activated myeloid cells promote release of nitric oxide (NO) through the activation of inducible NO synthase, iNOS (also known as NOS2), a hallmark of the classically activated pro‐inflammatory phenotype. We analyzed iNOS expression by FACS in AD models *vs* WT mice and in EAE *vs* controls mice. In iNOS population, we identified ‘iNOS^high^’ subpopulation which represented an extreme inflammatory state. A significant increase in iNOS and iNOS^high^ was observed in microglia and MDM in both AD models compared to age‐matched WT mice (Fig. 5A–D). In contrast, in EAE, MDM but not microglia exhibited an increase in iNOS expression (Fig. 5E, F), and iNOS^high^ expression level was not changed in MDM and microglia during EAE. We observed that iNOS expression level was increased in microglia from 24‐month‐old WT mice, while the percentage of iNOS‐expressing microglia was decreased (Fig. 5B).

We also analyzed the expression of iNOS by immunochemistry in the cortex sections from TgAPP/PS1 outside *vs* around Aβ plaques compared to age‐matched WT mice (Fig. 5G). We quantified iNOS expression colocalized with Iba1 marker, associated or not with Aβ plaques. We observed an increase in iNOS area around Aβ plaques in TgAPP/PS1 compared to WT mice (Fig. 5G), and iNOS expression is excluded from the core of the plaques like Iba1 staining (Figs. S1C and D and S6C). We wanted to characterize the iNOS expression by myeloid cells outside *vs* around Aβ plaques in a same brain section from TgAPP/PS1 mice. Interestingly, iNOS expression was increased in myeloid cells located in periplaques when compared to those outside plaques suggesting a local activation of myeloid cells rather than an effect due to diffusion of inflammatory factors in the brain parenchyma (Fig. 5G).

**Figure 6:**
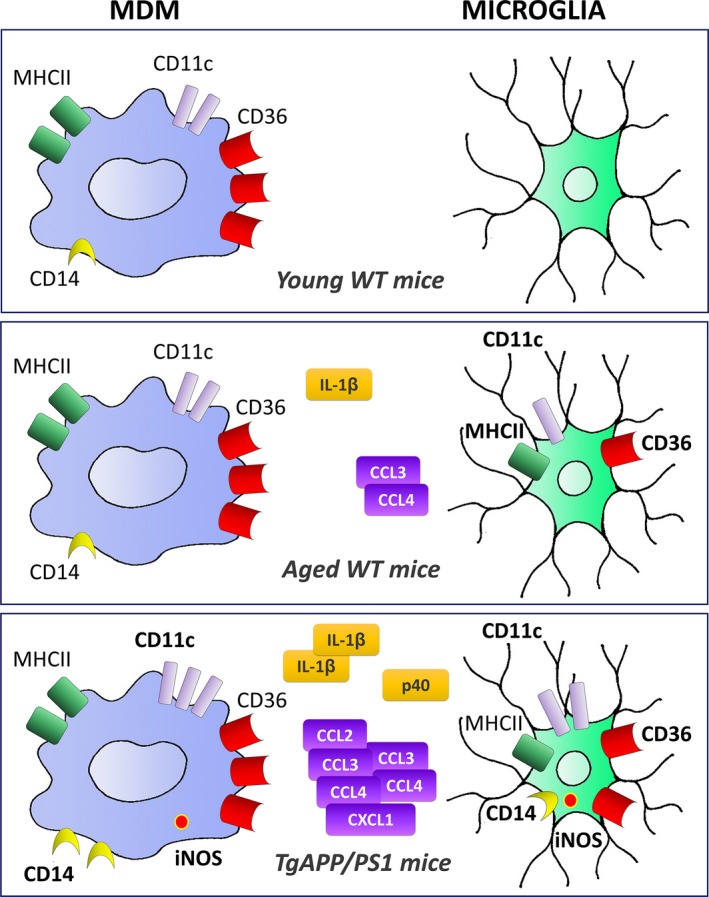
Schematic representation of specific microglia and MDM activation states in aged mice and AD model. MDM already present an activated phenotype in young mice and respond to Aβ peptides but are not affected by normal aging. In contrast, microglia express activation markers with normal aging and express several pro‐inflammatory mediators in specific response to Aβ peptides. Main cell surface markers, cytokines, and chemokines are depicted. Cell surface expression of MHCII, CD11c, CD36, and CD14 was assessed by flow cytometry. p40 expression was determined by flow cytometry. iNOS expression was measured by flow cytometry and immunohistochemistry. IL‐1β, CCL‐2, CCL‐3, CCL‐4, and CXCL‐1 expressions were quantified by ELISA and immunohistochemistry. Increased protein expression is indicated in bold.

Therefore, we observe a strong increase in iNOS in MDM and microglia at the stage of compact and very diffuse plaque burden of the pathology. Upregulation of iNOS is mainly associated with myeloid cells located in proximity to Aβ plaques (Fig. 6).

## Discussion

The precise functions of microglia and MDM in AD are still not clearly established. Experimental models indicated that MDM seem more efficient in AD, while microglia appear paralyzed (Simard *et al*., 2006; Grathwohl *et al*., 2009; Heneka *et al*., 2010). In our study, we aimed to 1) determine whether MDM infiltrate the CNS in AD mice without experimental manipulation, 2) highlight the specific inflammatory responses throughout Aβ pathology compared to normal aging, and 3) identify beneficial and pathogenic phenotypes of myeloid cells. Our data indicated MDM infiltration during normal aging and only at late stage of Aβ pathology. We determined that MDM presented an activated phenotype at basal state (Fig. 6). And, we showed that Aβ response differs from normal aging processes by a sustained production of pro‐inflammatory mediators (Fig. 6).

With aging, we observed in WT mice an increased expression of activation markers (CD11c, MHCII) as well as the receptor CD36 involved in phagocytosis (Fig. 6). This activation phenotype most likely reflects normal aging due to intrinsic aging processes or increasing levels of debris that need to be cleared up in the local environment (Conde & Streit, 2006; Mosher & Wyss‐Coray, 2014). Several works reported altered functions of microglia with aging such as slower process motilities and less migration (Damani *et al*., 2011; Baron *et al*., 2014). Thus, senescent microglia seem to be able to maintain the homeostasis of the brain but fail to respond to potential insults of the brain. We could hypothesize that aging microglia are overwhelmed and no longer able to regulate increasing amount of Aβ peptide accumulation. In addition to age effect, we showed that interactions between Aβ and microglia induced the release of pro‐inflammatory factors (Fig. 6). In aged TgAPP/PS1dE9 mice, increased expression of IL‐1β and TNF‐α by microglia was associated with lower phagocytic properties (Hickman *et al*., 2008; Krabbe *et al*., 2013; Babcock *et al*., 2015); thus, Aβ‐induced cytokine release may in turn lead to the alteration of microglial functions in a vicious circle.

The relative secretion levels of pro‐ and anti‐inflammatory factors can also provide an indication of the activation state of myeloid cells. Transcript levels partially reflect protein abundances (Vogel & Marcotte, 2012). Thus, we studied expression of inflammatory markers and mediators at the protein levels. We found consistent results with those determined at the mRNA level for increased expression of IL‐1β, TNF‐α, CCL‐3, and iNOS (Hickman *et al*., 2008; Jimenez *et al*., 2008; Minogue *et al*., 2014; Babcock *et al*., 2015), but we observed differences for IL‐4, IL‐10, and CD36 previously shown to be decreased (Hickman *et al*., 2008; Jimenez *et al*., 2008). In summary, we have evidenced a continuous increased production of inflammatory mediators IL‐1β, p40, iNOS, and chemokines in AD models (Fig. 6), but no anti‐inflammatory cytokines such as IL‐4 and IL‐10, indicating that microglia/MDM do not shift toward an alternative anti‐inflammatory phenotype during the pathology. This lack of transition could be explained by an effect of normal aging, as senescent microglia display a more sustained immune response that could not be downregulated (Damani *et al*., 2011; Harry, 2013).

We have observed that chemokines were already induced at the early stages of the disease in both AD models and were mostly produced by astrocytes highlighting their essential cross talk with microglia. Furman *et al*. have shown that inhibition of astrocyte activation by AAV reduced microglial activation in AD model (Furman *et al*., 2012). Thus, modulating astrocyte activation and their chemokine releases might be a new strategy in the treatment of AD.

We observed that a small population of CD11b^+^CD45^high^ cells was present in the brain of young WT mice which increases with age, and we found Iba1^+^CX3CR1^neg^ cells around vessels only in aged mice. Minogue *et al*. described blood–brain barrier disruption with age suggesting that MDM infiltration could be most likely an effect of aging (Minogue *et al*., 2014). This MDM infiltration is amplified in TgAPP/PS1 model, probably in response to CCL‐2 production observed at late stage of the disease. Our results are in line with the work of Mildner *et al*., showing that at early stages brain parenchyma was devoid of any engrafted peripheral cells in absence of brain irradiation (Mildner *et al*., 2011). Babcock *et al*. also demonstrated using bone marrow chimeric mice that the majority of myeloid cells were resident microglia (Babcock *et al*., 2015). Thus, we can hypothesize that MDM infiltration occurs too late to contribute efficiently to Aβ clearance. We observed that MDM are present around Aβ lesion before their localization around vessels in AD models. These results suggest that at early stage of Aβ pathology, MDM subpopulation seems mostly constituted of meningeal and choroid plexus macrophages (Aguzzi *et al*., 2013; Greter *et al*., 2015) rather than brain‐infiltrating macrophages as observed in EAE. CD11b^+^CD45^high^ cells are heterogeneous, notably expressing or not CCR2, and could reflect distinct differentiation stages as monocytes downregulate CCR2 upon differentiation (Greter *et al*., 2015). This limited subpopulation of MDM may have potential therapeutic value as several works have demonstrated the importance of CCR2 expression in AD models (El Khoury *et al*., 2007; Naert & Rivest, 2011). In another context, the CCR2^−^ subpopulation within the meninges rather than CCR2^+^ cells predominantly controls *Streptococcus pneumoniae* infection in the CNS (Mildner *et al*., 2008). Further analysis of these subpopulations is thus necessary to specifically target the appropriate population in AD.

In AD model, we assume that microglia are not properly activated to be functionally efficient, while MDM are able to restrict the formation of Aβ plaques (Simard *et al*., 2006). Our results showed that MDM constitutively expressed CD36 which can explain why MDM are more beneficial than microglia in AD. In EAE, microglia promote remyelination *via* phagocytosis of debris, and we observed that most microglia expressed CD36 (Yamasaki *et al*., 2014). Overall, high expression of CD36 is associated with beneficial phagocytosis suggesting that expression of CD36 should be improved in microglia. The coreceptor CD14 was upregulated in microglia and MDM specifically in response to Aβ peptides but not with age. Interestingly, we noted that although the percentage of CD14^high^ cells was increased in both models, it was tenfold higher in TgAPP/S1 mice *vs* TgAPP/PS1dE9. As CD14 specifically binds fibrillar Aβ42 (Fassbender *et al*., 2004), the difference between the two AD models could be explained by the higher ratio of Aβ42/Aβ40 in TgAPP/PS1 compared to TgAPP/PS1dE9 (ratio Aβ42/Aβ40 insoluble ≥ 15 for TgAPP/PS1 and ≥ 6 for TgAPP/PS1dE9, data not shown). Activation of CD14 and CD36 receptors should be induced with great care as phagocytosis of protein can result in the release of pro‐inflammatory cytokines such as IL‐1β (El Khoury *et al*., 2003; Sheedy *et al*., 2013). Nevertheless, treatment that induced upregulation of CD36 and the nonpyrogenic stimulation of CD14 have shown beneficial effects in AD models (Yamanaka *et al*., 2012; Michaud *et al*., 2013). These results indicate that Aβ peptides significantly influence CD14 and CD36 expression and these receptors are thus pertinent therapeutic targets (Fig. 6). We could hypothesize that the combined effects of aging and Aβ on CD36 stimulation induce a signal that leads to cytokines release. Aging‐dependent activation of CD36 could shift the CD14 pathway toward cytokine release in response to Aβ rather than phagocytosis. Another hypothesis is that phagocytosis of Aβ peptides by itself is not efficient and induces cytokine production via lysosomal damage and activation of the NLRP3 inflammasome (Halle *et al*., 2008; Sheedy *et al*., 2013). Signaling pathways activated by CD14 and CD36 need to be analyzed in depth to identify the checkpoint which leads to production of inflammatory factors *vs* efficient phagocytosis.

Our study identifies the specific phenotypes of microglia and MDM induced by normal aging or by accumulation of Aβ peptides. In our model (Fig. 6), we propose that Aβ pathology is mainly characterized by increased expression of pro‐inflammatory mediators, and we highlight the involvement of CD14 and CD36 in inflammatory processes. Thus, identifying signaling pathway elements that control phagocytosis without leading to release of inflammatory molecules constitutes pertinent therapeutic targets to improve the progression of AD.

## Experimental procedures

### Mice

APP/PS1dE9 mice were obtained from The Jackson Laboratory (number 005864) on the C57BL/6 background (Jankowsky *et al*., 2004), and APP/PS1 mice were obtained from M Jucker's Labs on the C57BL/6 background (Radde *et al*., 2006). Nontransgenic littermates were used as control mice. Mice were housed under conventional conditions at the housing facilities of ICM. Mice were used in accordance with the ARRIVE guidelines for care and use of experimental animals of the European Union.

### Isolation of brain myeloid cells and flow cytometry analyses

Preparation of CNS‐immune cells was performed using Percoll separation, and cells were labeled as described previously (Fazilleau *et al*., 2007). Detailed methods are included in Data S1. Fluorescence intensities were measured using a FACSVerse analyzer (BD Biosciences, Franklin Lakes, NJ, USA), and data were analyzed using the FlowJo Software (FlowJo LLC, Ashland, OR, USA).

### Induction and assessment of EAE

EAE was induced in 2‐month‐old C57BL/6 female mice as described previously (Delarasse *et al*., 2003). Detailed methods are included in Data S1.

### Tissue preparation

Mice were deeply anesthetized and transcardially perfused with 50 ml PBS. The brains were removed from the skull. One hemisphere was snap‐frozen for biochemical analysis, and the other was fixed in 4% paraformaldehyde and then frozen in dry ice and isopentane. Brain protein extraction. Snap‐frozen brain hemispheres were homogenized in tissue protein extraction reagent (T‐PER; Thermo Scientific) containing a mixture of protease and phosphatase inhibitors (Thermo Scientific). Homogenates were centrifuged at 100,000 g for 1 h at 4 °C. The supernatant was used for quantification of cytokines and chemokines.

### ELISA quantification of cerebral cytokines and chemokines

Quantitative determination of cerebral cytokines was performed using an electrochemiluminescence ELISA for IFN‐γ; IL‐1β; IL‐2; IL‐4; IL‐5; IL‐6; CXCL‐1; IL‐10; IL‐12p70; TNF‐α mouse pro‐inflammatory panel kit according to the manufacturer's guidelines (Meso Scale Discovery, Rockville, MD, USA). Signals were measured on a SECTOR Imager 2400 reader (Meso Scale Discovery). Quantitative determination of cerebral CCL‐2, CCL‐3, and CCL‐4 chemokines was performed using BD Cytometric Bead Array Kit according to the manufacturer's guidelines (BD Biosciences). Data acquisition was performed using a FACSVerse analyzer (BD Biosciences). Fluorescence intensity of beads coated with chemokines coupled to phycoerythrin (PE) and samples was analyzed with BD FACSuite Software (BD Biosciences). Each sample was measured in duplicate.

Aβ plaque staining and immunohistochemistry were performed as detailed in Data S1.

### Quantification analysis

For ELISA, each sample was measured in duplicate and secreted cytokines and chemokines were normalized to total brain protein concentration evaluated by BCA assay. Animal number was *n* = 4‐6 per genotype and age. For histology, images were quantified for labeled area fraction (%) by automated counting using ImageJ software. Briefly, images were normalized by subtracting background and an automatic thresholding (MaxEntropy) was applied. Mean values of area fraction (%) were obtained from images (*n* = 10) of cortex from AD models (*n* = 5) *vs* wild‐type (WT) mice (*n* = 5). The area of iNOS overlaying Iba1 in WT mice and in AD models outside *vs* around Aβ plaques was determined with Just Another Colocalization Plugin (JACoP) in ImageJ software. Per animal, a coverage of 75–200 plaques was determined.

### Statistical analysis

GraphPad Prism software (La Jolla, CA, USA) was used for statistical analyses. All data are expressed as mean ± SEM, and the mean significant difference between experimental groups was determined with one‐way analysis of variance (ANOVA) followed by Bonferroni's post hoc test. Alternatively, for comparisons between two groups, the mean significant difference was determined with a two‐tailed Student's unpaired *t*‐test. *P*‐values in graphic are represented as follows: *P < 0.05, ** *P* < 0.01, and *** *P* < 0.001.

## Funding

This work was supported by grants from Agence Nationale pour la Recherche (ANR‐12‐MALZ‐0003‐02‐P2X7RAD), Association France Alzheimer, and Bpifrance.

## Conflict of interests

None declared.

## Author contributions

E.M. and C.D. designed the project. E.M., C.B., and C.D. performed the research. E.M., C.B., B.F., and C.D. analyzed the data. E.M., B.F., and C.D. wrote the manuscript.

## Supporting information


**Fig. S1** Analyses of Aβ plaques load and myeloid cells in AD models.
**Fig. S2** Analyses of microglia and MDM localization in AD models and EAE mice.
**Fig. S3** Analyses of cytokines expression in AD models and EAE mice by FACS.
**Fig. S4** Analyses of cytokines levels in AD models by multiplex ELISA.
**Fig. S5** Analyses of cell specific expression of chemokines in AD model.
**Fig. S6** Analyses of myeloid cell morphology in AD model.
**Data S1** Experimental procedures.
