# Supplementary material for: Distinct inflammatory phenotypes of microglia and monocyte‐derived macrophages in Alzheimer's disease models: effects of aging and amyloid pathology
Source: Aging Cell. 2016 Oct 8;16(1):27–38. doi: 10.1111/acel.12522 (PMC5242297; doi:10.1111/acel.12522)
Supplement: Supplementary file 1 — Fig. S1 Analyses of Aβ plaques load and myeloid cells in AD models. Fig. S2 Analyses of microglia and MDM localization in AD models and EAE mice. Fig. S3 Analyses of cytokines expression in AD models and EAE mice by FACS. Fig. S4 Analyses of cytokines levels in AD models by multiplex ELISA. Fig. S5 Analyses of cell specific expression of chemokines in AD model. Fig. S6 Analyses of myeloid cell morphology in AD model. Data S1 Experimental procedures. [file ACEL-16-27-s001.pdf]

Supporting Information Martin *et al.*

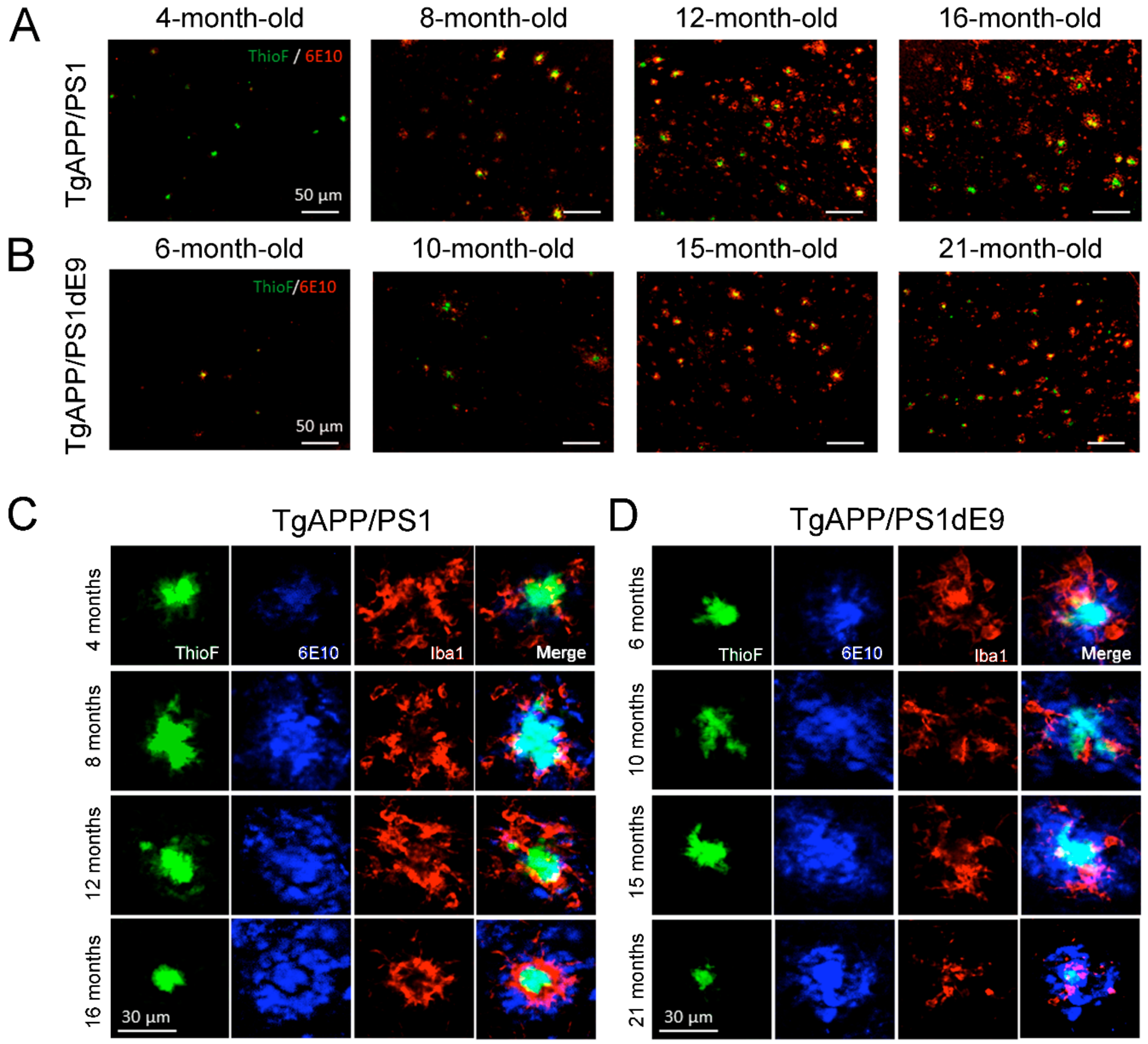

Supplemental Fig 1. Analyses of A_ plaques load and myeloid cells in AD models. Amyloid plaque load was assessed by Thioflavin S staining and A_ peptides with 6E10 antibody in TgAPP/PS1 mice at 4, 8, 12 and 16 months (A) and in TgAPP/PS1dE9 at 6, 10, 16 and 21 months (B). Myeloid cell localization in dense-core plaques (Thioflavin-labeled core and 6E10-labeled halo) was assessed by Iba1 antibody in TgAPP/PS1 mice at 4, 8, 12 and 16 months (C) and in TgAPP/PS1dE9 at 6, 10, 16 and 21 months (D).

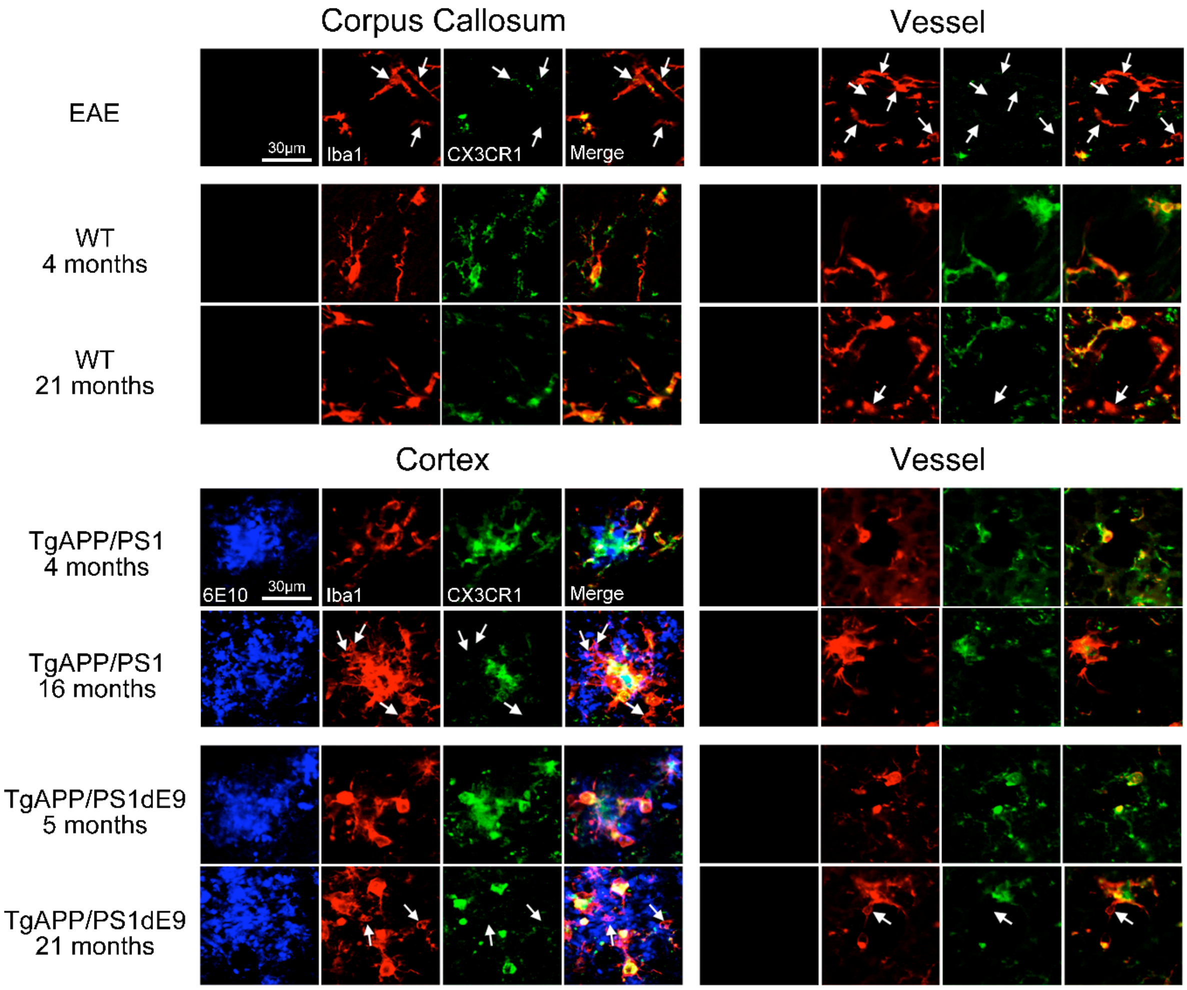

Supplemental Fig 2. Analyses of microglia and MDM localization in AD models and EAE mice. The localization of microglia and MDM were assessed by immunohistochemistry in the brain of young 4-month-old WT mice, aged 24-month-old mice, 2-month-old EAE mice, 4- and 16-month-old TgAPP/PS1 mice and 5- and 21-month-old TgAPP/PS1dE9 mice. Myeloid cells were labelled with anti-Iba1 antibody, microglia with anti-CX3CR1 antibody and amyloid plaques with anti-A_ antibody (6E10). MDM cells are indicated by an arrow.

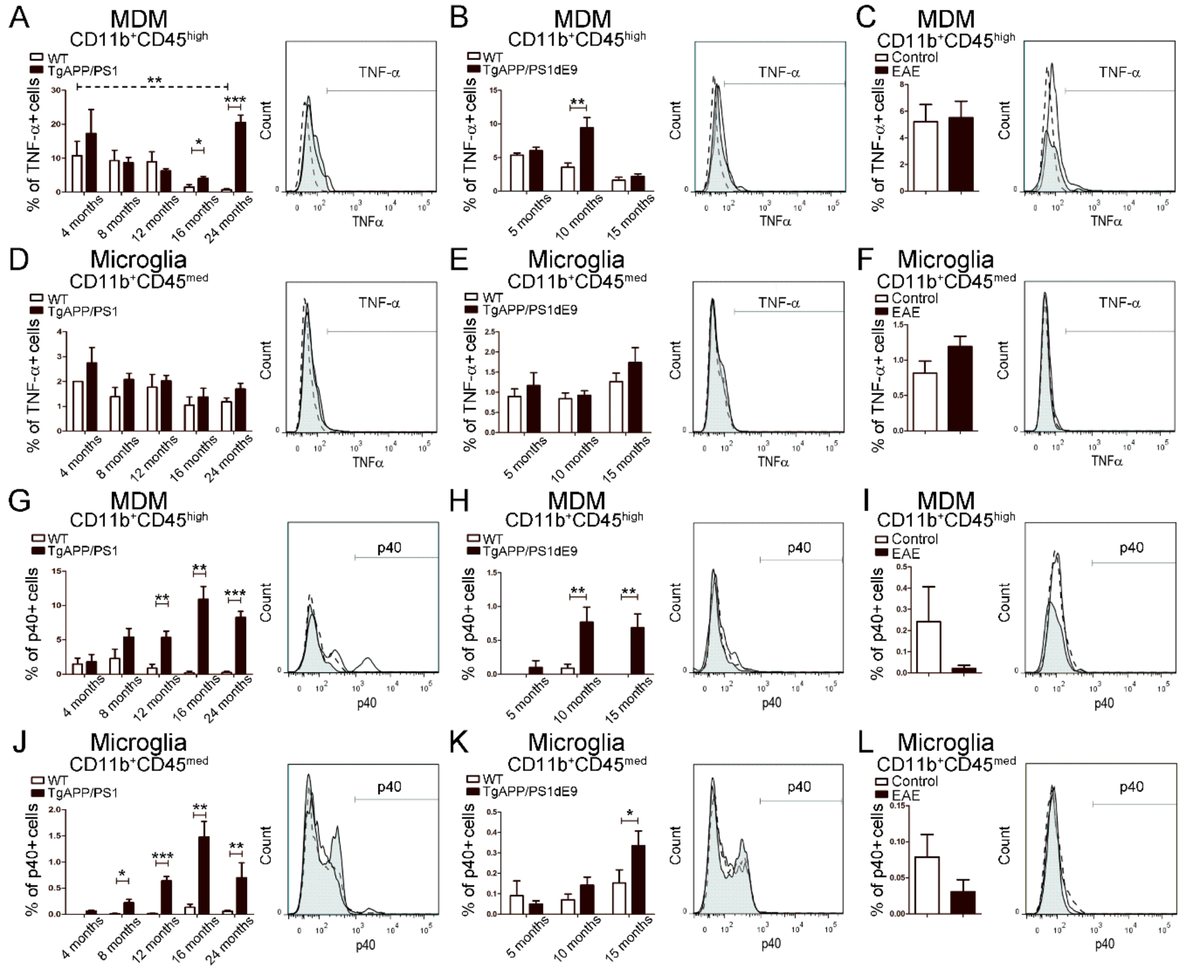

Supplemental Fig 3. Analyses of cytokines expression in AD models and EAE mice by FACS. TNF_ (A-F) and p40 (G-L) expression on $CD11b^+CD45^{high}$ MDM populations (A-C and G-I) and $CD11b^+CD45^{med}$ microglia (D-F and J-L) from WT littermate controls, TgAPP/PS1 mice (A, D, G, J) (4 months (n=4), 8 months (n=6), 12 months (n=9), 16 months (n=5) & 24 months (n=4) and TgAPP/PS1dE9 mice (B, E, H, K) (5 months (n=8), 10 months (n=10) & 15 months (n=8) and EAE mice, score 3 (C, F, I, L) (2 months n=9). Gates shown in the FACS histograms (right panel) illustrate TNF_ (A-F) and p40 (G-L) expression in $CD11b^+CD45^{high}$ cells and $CD11b^+CD45^{med}$ cells from WT mice (Black histogram) and 16-month-old TgAPP/PS1 mice (A, D, G, J), 15-month-old TgAPP/PS1dE9 mice (B, E, H, K) and 2-month-old EAE mice, score 3 (C, F, I, L) (black tinted histogram). Isotype antibodies were used as negative controls (dotted histogram). Data are expressed as means ± SEM. * $p<0.05$, ** $p<0.01$, *** $p<0.001$ statistical significance.

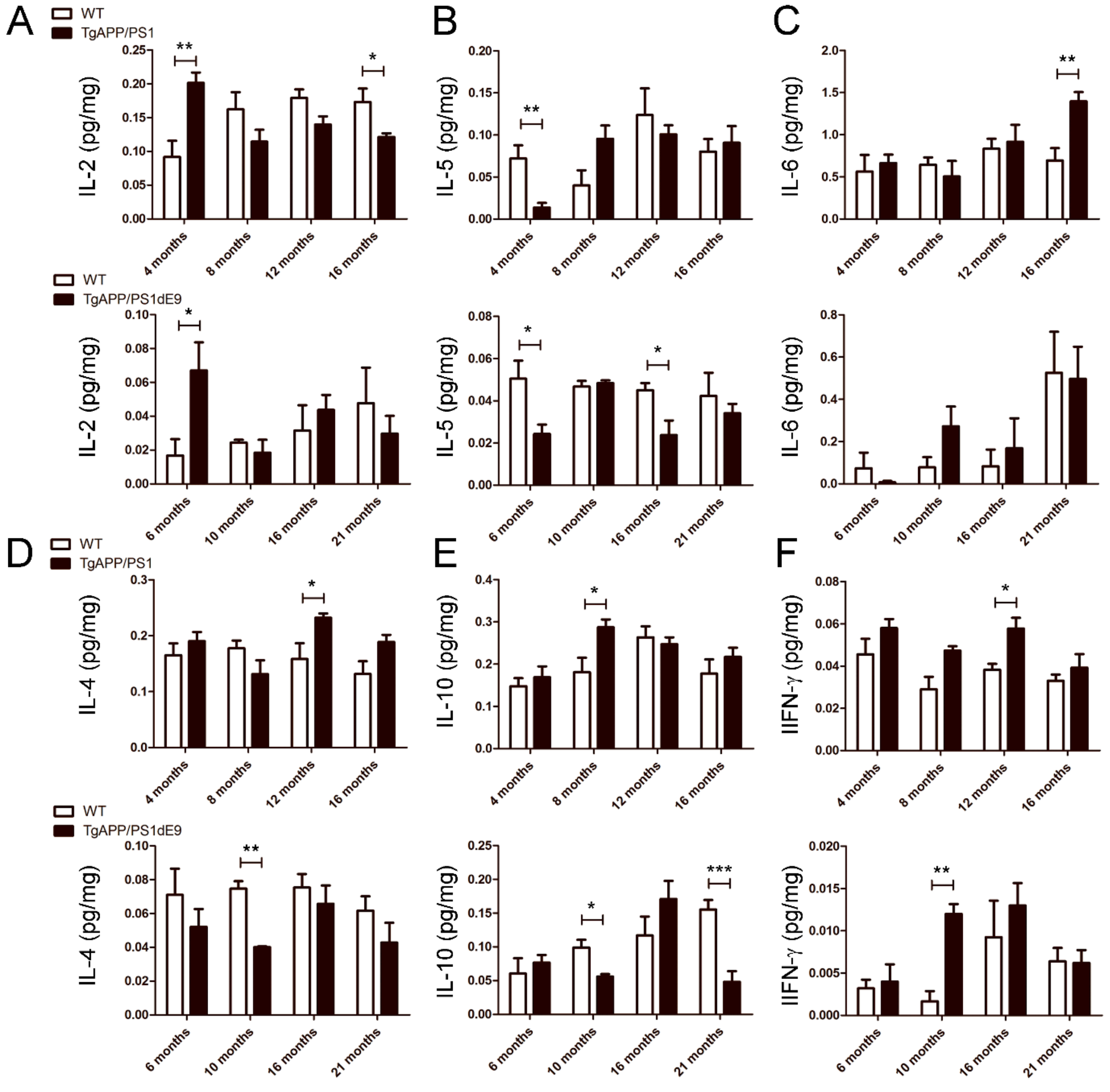

Supplemental Fig. 4. Analyses of cytokines levels in AD models by multiplex ELISA. IL-2 (A), IL-5 (B), IL-6 (C), IL-4 (D), IL-10 (E) and IFN-_ (F) protein expression levels were determined by multiplex ELISA in brain of TgAPP/PS1 mice (4 months, 8 months, 12 months & 16 months; n=4-6 per group) (upper panel) and of TgAPP/PS1dE9 mice (6 months, 10 months, 16 months & 21 months; n=4-6 per group) (lower panel) and age-matched WT littermate controls (n=4-6 per group). Data are expressed as means ± SEM. * p<0.05, ** p<0.01, *** p<0.001 statistical significance.

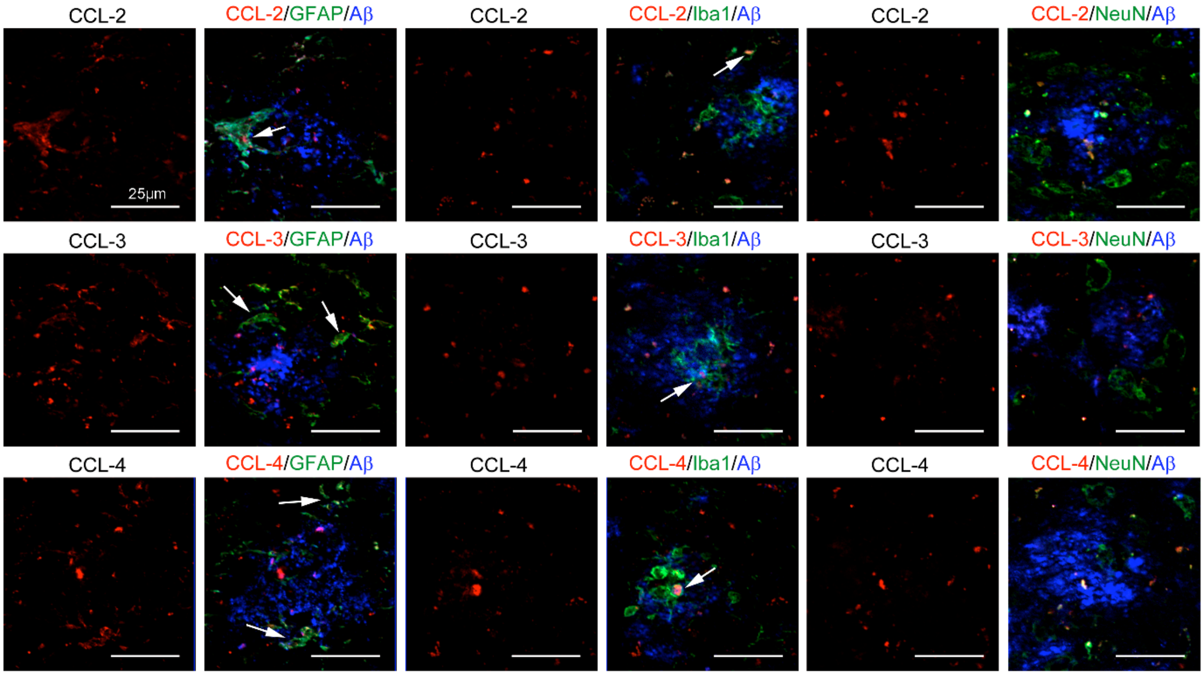

Supplemental Figure 5. Analyses of cell specific expression of chemokines in AD model. The expression of CCL-2, CCL-3 and CCL-4 were assessed by immunohistochemistry in the brain of 12-month-old TgAPP/PS1 mice. Amyloid plaques were labelled with anti-A_ antibody (4G8), astrocytes with anti-GFAP antibody, myeloid cells with anti-Iba1 antibody and neurons with anti-NeuN antibody. Arrows indicate double immunostaining.

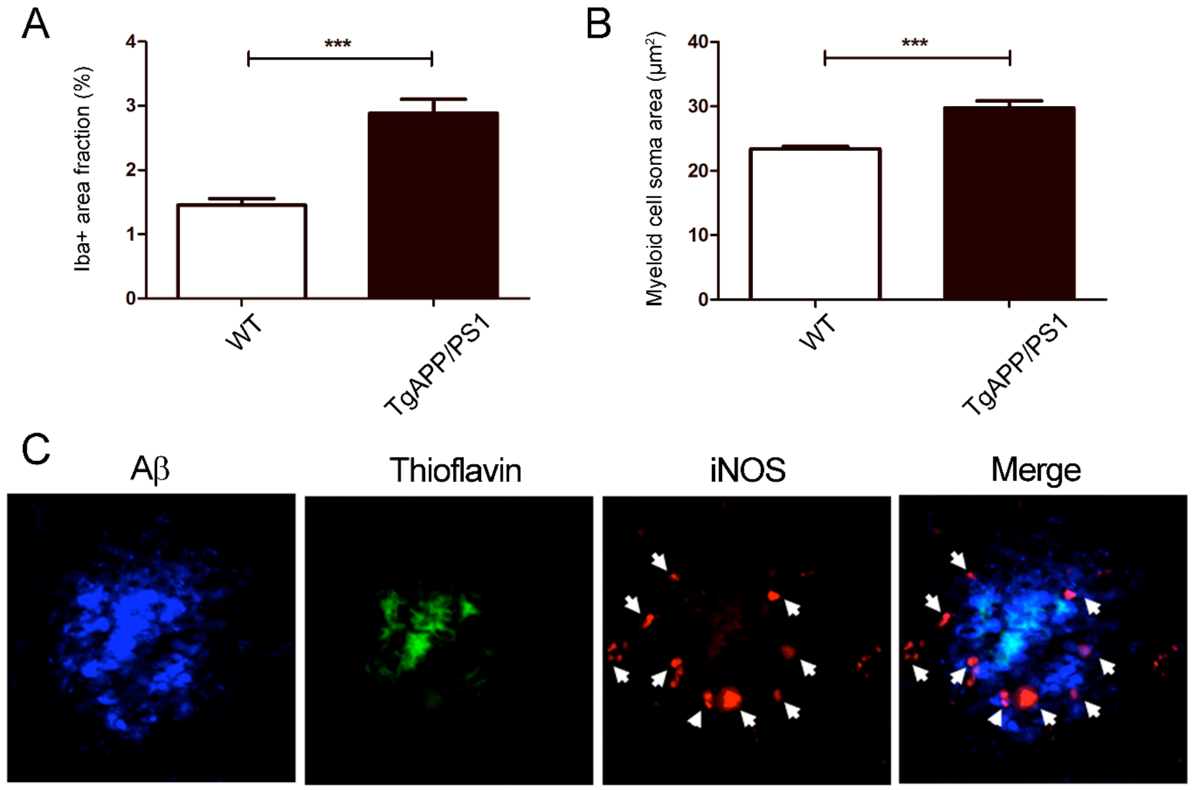

Supplemental Figure 6. Analyses of myeloid cell morphology in AD model. Myeloid cells from brain sections of 16-month-old WT and TgAPP/PS1 mice were stained with Iba1 antibody. Quantification of surface area of iba1 staining on total surface was performed in 10 consecutive sections per animal and is given as % of area fraction (n=5 per group) (A). Myeloid cell soma area was determined using ImageJ software. Data are expressed as means ± SEM. *** $p<0.001$ statistical significance. Brain sections of 16-month-old WT and TgAPP/PS1 mice were stained for iNOS (red), Thioflavin S staining (green) and A_ peptides with 6E10 antibody (blue) (C).

Supplementary Experimental Procedures

Induction and assessment of EAE

C57BL/6 females were immunized subcutaneously with 200 µg MOG35–55 (MEVGWYRSPFSRVVHLYRNGK), in CFA containing 500 µg of Mycobacterium tuberculosis H37RA (Difco Laboratories, Detroit, MI) as described previously (Delarasse et al. 2003). Mice received intravenous injection of 200 ng of pertussis toxin (List Biological Laboratories Inc., Campbell, CA) on days 0 and 2. Clinical signs of EAE were assessed daily with a 0–5 scoring range (0, healthy; 1, flaccid tail; 2, impaired righting reflex and/or abnormal gait; 3, partial hind leg paralysis; 4, total hind leg paralysis; 5, hind leg paralysis with partial front leg paralysis).

Isolation of brain-myeloid cells and flow cytometry analyses

Preparation of CNS-immune cells was performed using Percoll separation as described previously. Briefly, mice were perfused with phosphate-buffered saline (PBS) and the brains were dissected out and homogenized in 5 ml of PBS with collagenase IV at 0.2 mg/ml and DNase I at 1 mg/ml (both from Sigma-Aldrich) for 30 min at 37°C and 5% CO2. The brain homogenate was washed with PBS-FBS 5% and centrifuged in a 30/37/70% Percoll gradient (GE Healthcare). Myeloid cells were recovered from the 37/70% Percoll interphase and washed. The pellet containing the myeloid cells was re-suspended in 400 µl PBS-BSA 0.5%. Binding of antibodies to Fc receptors was prevented by adding 1 µg Fc-block (CD16/CD32 Abs). 100 µL of cells were labelled with 100 µl of primary antibodies mix: anti-CD45-V450 (30F11), anti-CD11b-PerCPCy5.5 (M1/70), Gr1-FITC (RB6-8C5), anti-CD14-FITC (Sa2-8),

MHCII-PE (M5/114.15.2), anti-p40-PE (C17.8), anti-iNOS-FITC (clone 6), anti-CD11c-APC (N418), anti-CD36-APC (72-1), anti-TNF-_-APC (MP6-XT22)(BD Biosciences and eBioscience), anti-CX3CR1-PE, anti-CCR2-APC (R&D systems). For intracellular staining, cells were permeabilized with PBS-BSA 0.5%-Saponin 0.05%. After labeling cells were fixed in 1% paraformaldehyde (PFA). A logical gate strategy was applied for the FACs experiments: a morphological gating referred to dot plot FSC-Area versus SSC-Area and duplets discrimination referred to dot plot FSC-Area versus FSC-High. To standardize our FACS analyzes, we have analyzed the same number of cells for each sample i.e. 20 000 events in this gate per sample. For compensation settings, compbeads (BD Biosciences) with corresponding antibodies were used. Isotype-matched control antibodies were used as negative controls. Fluorescence intensities were measured using a FACSVerse analyzer (BD Biosciences) and data were analyzed using the FlowJo Software.

A_ plaques staining and immunohistochemistry

20 µm serial sections were cut with cryostat (Leica, Wetzlar, Germany). Sections were stored at -20°C. For plaques histology, sections were stained with 0.01% thioflavinS (Sigma), PBS for 5 min at room temperature, cleared in 70% ethanol for 5 min and then washed 3 times in PBS. For immunohistochemistry, sections were incubated in 0.2% Triton X-100, PBS for 15 min. Then, sections were incubated in 3% BSA, 0.05% Triton_X-100, PBS (blocking solution) for 45 min followed by overnight incubation at +4°C with the primary antibody in blocking solution. The following primary antibodies were used with respective concentrations: rabbit polyclonal anti-GFAP (1:2000, Z0334, DAKO, Ely, Cambridgeshire, UK), rabbit polyclonal anti-Iba1 (1:2000, 019-19741, Wako Chemicals GmbH, Neuss, Germany), mouse monoclonal anti-NeuN (1:200, MAB377, Merck Millipore, Darmstadt,

Germany), mouse monoclonal anti-iNosII (1:100, 610329, BD Transduction Laboratories), mouse monoclonal anti-_-amyloid, 1-16 (6E10) (1:2000, SIG-39320, Covance), mouse monoclonal anti-_-amyloid, 17-24 (4G8) (1:2000, SIG-39220, Covance), rabbit polyclonal anti-IL-1_ (1:100, ab9722, Abcam, Cambridge, United Kingdom), goat polyclonal anti-CX3CR1-PE (1:20, R&D systems). Alternatively, for CCL-2, CCL-3 and CCL-4 immunostaining, sections were incubated in 0.1% Triton X-100 then blocked in 4% BSA, 0.4% Triton_X-100, PBS and incubated with primary antibody against goat polyclonal anti-CCL-2 (1:200, AF-479-NA, R&D Systems, Minneapolis MN, USA), goat polyclonal anti-CCL-3 (1:200, AF-450-NA, R&D Systems), and goat polyclonal anti-CCL-4 (1:200, AF-451-NA, R&D Systems) in 0.1% Triton X-100, PBS for two days. Sections were washed three times in 0.05% Triton_X-100, PBS and incubated with Alexa-488- or Alexa-594- or Alexa-647-conjugated secondary antibodies (1:1000, Invitrogen) for 90_ min, washed 3 times with 0.05% Triton X-100, PBS for 5_ min. Then, sections were mounted using Fluoromount-G (17984-25, Electron Microscopy Sciences). Fluorescence microscopy was performed on a Zeiss AxioImager.M2 microscope equipped with an apotome system. Images were processed as stacks of 5 _m with 0.5 _m between sections. Z-projections and orthogonal projections were done with Zeiss Zen 2 software.
